# Critique of autonomy‐based arguments against legalising assisted dying

**DOI:** 10.1111/bioe.13125

**Published:** 2022-11-23

**Authors:** Thomas Søbirk Petersen, Morten Dige

**Affiliations:** ^1^ Department for Communication and Arts Roskilde Universitet Institut for Kultur og Identitet Roskilde Denmark; ^2^ Department of Philosophy and History of Ideas Aarhus University Aarhus Denmark

**Keywords:** assisted dying, autonomy, ethics, pain, terminally ill

## Abstract

The aim of this article is to present and critically investigate a type of argument against legalising assisted dying on request (ADR) for patients who are terminally ill and experiencing suffering. This type of argument has several variants. These—which we call ‘autonomy‐based arguments’ against legalising ADR—invoke different specifications of the premise that we ought not to respect requests for assistance in dying made by terminally ill and suffering patients because the basic conditions of autonomy cannot be met in scenarios where such requests are made. Specifically, it is argued either (1) that as a result of pain, anxiety or desperation, terminally ill patients are not competent decision makers or (2) that legalisation of ADR would lead to social pressure or in other ways change the patient's context of choice in ways that make such requests nonautonomous. We argue that these types of arguments are problematic in light both of empirical studies and the fact that we usually judge that it is morally right to respect the wishes and decisions of dying people even if they suffer.

## 1 INTRODUCTION

The aim of this article is to present and critically investigate a type of argument against legalising assisted dying on request (henceforth, ADR) for patients who are terminally ill and experiencing suffering. This type of argument has several variants. These—which we call ‘autonomy‐based arguments’ against legalising ADR—invoke different specifications of the premise that we ought not to respect requests for assistance in dying made by terminally ill and suffering patients because the basic conditions of autonomy cannot be met in scenarios where such requests are made. Specifically, it is argued either (1) that because of pain, anxiety or desperation, terminally ill patients are not competent decision makers or (2) that legalisation of ADR would lead to social pressure or in other ways change the patient's context of choice in ways that make such requests nonautonomous. In other words, when terminally ill patients who experience suffering make a request for assisted dying they are either not competent, and therefore, there is no autonomy to respect,^1^ or the mere legalisation of ADR would change the situation in ways that are incompatible with the patient making an autonomous choice. For example, by creating undue social pressure on the patient.^2^ We suggest that the overall structure of these arguments can be reconstructed as follows.

### 1.1 Overall autonomy‐based argument against ADR

P1: If patients who request assisted dying are not autonomous, assisted dying should not be legalised.

P2: Patients who request assisted dying are not autonomous.

Conclusion: Assisted dying should not be legal.

As the argument is valid, our critical discussion will focus primarily on P2. There has been some criticism of premises like P1 in arguments such as the above. This criticism centres on ethical concerns about patients who are not autonomous because they are not competent decision‐makers. For example, it has been asked whether children, or people suffering from dementia or severe mental illness, should be assisted to die even though they are not autonomous.^3^ However interesting and important these discussions are, they are not what we want to examine in this article. We want to examine autonomy‐based arguments against ADR. Therefore, for now, we shall accept premise P1.

However, before we move on to a critical discussion of P2, we shall first make some comments on the central concept of autonomy. Autonomy is usually understood as self‐determination or self‐governance. In other words, acting autonomously means acting in accordance with one's own values, reasons and motives. Therefore, when it comes to end‐of‐life decisions, autonomy means the capacity to act in accordance with one's own reasons and values with regard to one's own death. Respect for autonomy is clearly important, whether it is understood as an intrinsic or an instrumental value. The extent to which people can influence and control their own lives matters a great deal to them. However, to evaluate the argument, we need to know when it is true that a person is making an autonomous choice. In what follows, it is presumed that for an individual to make an autonomous choice, it is required that the agent must: (a) have access to information about relevant and available options and the expected risks and benefits of these options, (b) have the ability to understand this information, to form preferences about the options and to make a choice based on the information and any relevant related preferences of the agent and finally (c) be deciding in a context in which the agent is free from undue influence such as coercion, manipulation, pressure and social conditioning.^4^ These three requirements are what many moral philosophers would accept as necessary requirements, which must be complied with in order to make an autonomous choice.^5^ That is, if any of these requirements are not satisfied, an autonomous choice is impossible. We will refer to them as respectively (a) the information, (b) the competence and (c) the freedom requirement, and we will focus on the final two since they have been challenged in recent contributions to the debate over ADR.

The importance of this article is twofold. First, it is beyond doubt that many proponents of the legalisation of some forms of ADR justify their view by arguing that legalisation follows from respect for the autonomy of the dying.^6^ Therefore, if autonomy is not possible, or cannot be respected, if ADR is legalised, proponents of legalisation have lost one of their central arguments in favour of ADR.^7^ Second, apart from a few articles mentioning or presenting these autonomy‐based arguments against the legalisation of ADR, few scholars have critically discussed the variants of the argument mentioned above in detail.

In the next section, before we critically investigate two variants of the overall autonomy‐based argument against the legalisation of ADR in Sections 3 and 4, we want to say a few words about what we have in mind when we talk about ADR. Section 5 sums up the findings.

## 2 WHAT IS ADR?

As there is no clear consensus on the terminology of assisted dying, let us make a few specifications. In what follows, we take ADR to be a request for help to die. Our discussion covers two types of assisted dying. First, assisted dying can be realised by means of what is often called physician‐assisted suicide in which a physician delivers the knowledge and/or medication that will make it possible for the patient to end their life. Second, assisted dying can be performed directly by the physician (by administrating a lethal injection), perhaps because the patient in question is too weak to do so. Countries or states that accept ADR generally do so based on the fulfilment of some (but not all) of the following five conditions: (1) the patient is terminally ill; (2) there is no cure for the disease; (3) the patient is suffering unbearably without the prospect of improvement; (4) the patient is a competent decision‐maker and (5) the request is made voluntarily. Much could and has been said about how to specify these five conditions and whether we should accept all of them.^8^ However, to specify and critically discuss all five conditions in detail is impossible in such a short article. In the next and more critical section of the paper, we will therefore only specify and discuss in detail the last two conditions, both of which are essential for autonomous choice.

## 3 ARE DYING PEOPLE IN PAIN COMPETENT DECISION‐MAKERS?

As a point of departure, we would like to quote comments made by proponents of the claim that it is an illusion to imagine that we can respect the autonomy of terminally ill patients in pain who request assistance to die. Thus, Neil Campbell believes that it is not possible for a person in unbearable pain to decide voluntarily to have his or her life ended. ‘If the pain and suffering are by definition unbearable, then it seems clear enough that the decision to die is not freely chosen but is compelled by the pain’.^9^ According to Campbell, this observation makes it obvious that a request to end one's life when one is in unbearable pain is not made freely and should therefore not be acted on. Before we move on to a detailed critical discussion of this kind of view, we should note that Campbell's way of thinking is unclear. It sounds strange to claim, as Campbell does, that I cannot choose freely in a situation where a choice is compelled by pain. For example, imagine being in labour and in unbearable pain. In such a situation, it seems right to claim that you can still freely choose what kind of assistance you want: whether you want your partner to be present, whether you want extra oxygen, and so forth. Even though Campbell does not mention legalisation (stating only that it would be ‘… inadvisable for physicians to act in accordance with…’ the patient's wish to die), his way of thinking seems to provide an argument against the legalisation of ADR as well.^10^

Another example of the idea that autonomy is as an illusion when a terminally ill patient wants assistance to die can be found in the recent monograph by Ole Hartling:

It is forgotten that the decision—the decision about one's own death—is not made in a day‐to‐day context … It is a wish that arises against a backdrop of desperation and a feeling of hopelessness, and possibly a feeling of being superfluous.^11^

The focus here, as compared with the quotes by Campbell, is not necessarily that the pain is unbearable, but that the mental suffering is sufficient to make autonomous decision‐making impossible. Although these kinds of argumentation, based on the illusion of autonomy, do not specify which of the three requirements of autonomy cause the illusion, it seems fair to say that the above remarks focus on the competence requirement. So let us now turn our attention to a critical discussion of such versions of the overall autonomy‐based argument against ADR.

Should we accept these versions of P2 in the overall autonomy‐based argument against ADR? Is it true, or fair to say, that patients requesting assistance to die, even though they are suffering unbearable pain either physically or mentally, should most of the time, or always, be thought of as noncompetent? In what follows, we give five reasons for thinking that the answer is: not necessarily.

First, physical pain is rarely the primary problem for terminally ill patients. Modern palliative care is generally a success story, and patients requesting assisted dying state reasons like ‘loss of dignity’ and the ‘undignified process of dying’ far more often than they refer to the pain in and of itself.^12^ The final stages of certain chronic degenerative diseases, such as amyotrophic lateral sclerosis, involve extreme physical and/or mental deterioration that for many patients amounts to a wholly unacceptable and unbearable kind of mental suffering even if sufficient pain control is given. The picture of a terminally ill patient requesting assisted dying while writhing in pain or being desperate does not represent typical ADR scenarios. Campbell and Hartling provide no evidence that patients requesting assisted dying are in unbearable pain or that they are desperate or feeling hopeless.

Second, even if pain were the reason for requesting assisted dying, it would be reasonable to point out that the patient does not suffer unbearable pain all the time, either because the palliative care is working from time to time or because the pain is being paused by biological causes. In other words, the unbearable pain may come and go and may therefore change from day to day or from one hour to the next.^13^ However, if there are some breaks in the experience of pain, periods will exist in which patients may very well have the mental capacity to understand the relevant information about their situation and to evaluate what it is best for them to do, thus being competent decision‐makers.

Third, it seems obvious that we should at least sometimes accept that the request to die from people dying of unbearable pain could meet the competence requirement. Imagine a patient suffering intense pain caused by severe tongue cancer. Minutes before the patient is about to suffocate because of the growing and swollen tongue, the patient, who is obviously in physical pain and emotional distress, begs a physician to end her life before she is choked. Although the patient suffers from unbearable pain and is highly uncomfortable with the situation, it seems correct to say that she has access to the relevant information about her situation, together with the ability to form preferences about the options and make a choice on that basis. Whether or not it is true that you can be in pain but still be able to understand and process information, which is relevant to your life, is largely an empirical matter, it just seems far from obvious that the answer must be negative.

Fourth, it is a very common and morally acceptable practice, in a hospital setting, for the staff to accept decisions and thereby respect the autonomy of patients who have unbearable pain or who are in a hopeless or desperate situation. Terminally ill patients who suffer from unbearable pain and are desperate about their prospects often reject an operation or a treatment that will prolong their lives. Doctors usually respect the choices of such patients unless they are children or are otherwise not competent decision‐makers (e.g., have severe dementia). In addition, doctors will accord this respect even to patients whose decisions will make it harder to control their pain. In fact, there are situations in which requests for assisted dying by terminally ill patients are protected by law in many countries, such as when such patients no longer want to eat or drink. Moreover, some of the consequences of refusing requests for assisted dying made by the terminally ill on grounds of noncompetence speak in favour of not accepting certain forms of passive euthanasia, such as removing a respirator at the patient's request, either. Proponents of autonomy‐based arguments against ADR do not seem to be aware of this, as they are typically in favour of this type of passive euthanasia.^14^ So, in a nutshell, the problem is that if we already accept that we ought to respect the autonomy of people who suffer elsewhere in our healthcare system, then why should we not do the same when it comes to ADR?^15^

Finally, certain consequences of not respecting requests for assisted dying made by terminally ill people seem even more problematic. For it seems to follow from the idea that terminally ill patients suffering from unbearable pain are not competent and that we should not accept other wishes such dying patients may have either. For example, consider circumstances in which they want to make a will, change one, die at home, have their funeral at a specific church, and so on. We would argue instead that this worry about the illusion of competence in end‐of‐life decisions, which of course can be applicable in certain cases, is furthermore mitigated by studies showing that people at the end of their lives are in fact capable of making competent decisions.^16^

## 4 ARE DYING PEOPLE FREE TO CHOOSE TO DIE?

Just as autonomy can be regarded as an illusion where the patient does not satisfy the competence requirement because they are suffering unbearably, it can also be seen as illusory when the patient's decision to die does not satisfy the freedom requirement. Below we present four ways in which the freedom requirement might be rejected and discuss each critically.

First, scholars have argued that legalising ADR would generate subtle (or not‐so‐subtle) pressure to choose it. It is a kind of pressure that is morally problematic, as it undermines the patient's autonomy even though they satisfy the information and the competence requirement. For instance, Iona Heath has argued:

It seems to me to be impossible to ensure that an apparently voluntary request for assisted dying is not in some small way coerced. It is all too easy for sick and disabled people to believe that they are becoming an intolerable burden to those closest to them, and indeed, they often are a burden. In such circumstances, a request for assisted dying can become a sort of sacrifice on the part of the dying person, with complicit, self‐interested support from relatives, professionals or carers.^17^

Consider also Hartling:

But if a law on assisted dying gives the patient a right to die, that right may turn into a duty to die. How autonomously can the weakest people act when the world around them deems their ill, dependent, and pained quality of life as beyond recovery?^18^

It is obvious that often we cannot be involved in decision‐making without the influence of other people's opinions. We discuss things with, and listen to, other people, and it is often difficult to know when and how a decision of one's own is influenced by others. The important thing, though, is whether that influence is coercive or otherwise morally problematic. We can acknowledge that terminally ill patients are in a vulnerable position in which undue influence is a potential risk. Presumably, such undue influence would particularly affect terminally ill patients in vulnerable groups (e.g., the elderly, people with low educational status, the physically disabled), leading to a higher incidence of ADR in such groups. Comprehensive data from Oregon and the Netherlands, however, have shown no evidence of disproportionate adoption of legal ADR by patients in vulnerable groups.^19^ We suggest that the explanation for this is straightforward: health professionals are strongly motivated to protect patients against undue influence. Strong protections against undue influence therefore represent a feasible alternative to prohibiting ADR altogether. It is worth noting that terminally ill patients may also come under pressure not to initiate, or to discontinue, life‐prolonging treatment for dubious economic reasons, fear of becoming an intolerable burden or ‘the notion that certain lives are not worth preserving’.^20^ But it seems an overreaction to deny terminally ill patients the right to say no to life‐prolonging treatment just to avoid that risk. We trust instead that the risk can be handled: that sometimes we do have valid reasons to believe that patients are not under such undue influence from others. It is hard to see why this could not also be the case with terminally ill patients who request assisted dying, especially if you already accept passive euthanasia.

Second, it has been argued that the legalisation of ADR would coerce terminally ill patients into making a decision either to request assisted death or not to do so. Being free *to* choose comes with the cost of not being free *from* choosing. This argument was made famous by Velleman.^21^ He pointed out that more choices do not necessarily enhance (positive) freedom. A mundane example is being asked out to dinner, which presents an option that we would sometimes rather be without. After the invitation you cannot *just* stay home, it will now be the result of a decision—that is, something that you could potentially be asked to justify. For the terminally ill, ‘the point is that the patient cannot get out of having to choose. It has been called “the prison of freedom”’.^22^ This objection, however, is based on the premise that we typically approach the end of life as individuals who are free from making the kind of decision involved in ADR. But in many cases, or even most, adding the option of ADR would *not* change the process of dying from a nondecision situation (in which ‘nature takes its course’ with no conscious choices being made by the patient or the patient's relatives) to a decision situation. ADR scenarios are, in that sense, not unique. They are typically scenarios in which decisions about the end of life, e.g. whether or not one wants to receive artificial hydration and nutrition, are called for anyway. Therefore, what we see is not a case of autonomy fanatics trying to create a situation of choice and forcing decisions where none were called for. Instead, we are considering merely whether we ought to honour a widespread wish that ADR should be among the options in end‐of‐life decision‐making.^23^

Third, some have argued that it is a mistake to suppose that we can exercise autonomy over our own deaths. Death is the one thing that is not an object of choice. Hartling points out (correctly): ‘Autonomy with respect to your own death, however, is already halved: you can choose to die if you don't want to live, but you cannot choose to live if you are about to die’.^24^ Hartling makes it appear that proponents of ADR are under the illusion that we can gain total control over our own death. We fail to see, however, that mainstream autonomy‐based arguments in favour of the legalisation of ADR involve that illusion. We should be clear that legalised ADR does not involve a *right to choose death* (let alone a right to choose not to die, which, if permanent, would be a form of immortality) but a *right to choose the manner of one's death*. Further, we must stress that what such a right involves is not even a free (unlimited, unrestricted) choice of the manner of one's death, but simply a choice in which ADR, under strict conditions and regulations, is among the options.

Finally, it has been claimed that it is self‐defeating to respect autonomy by eradicating it by causing death. Initially, it might seem paradoxical to respect a person's right to autonomy if the result is that autonomy for this person is thereby ruled out forever: ‘And those around you must respect the right to self‐determination. The respect refers to a person who is respected, but this is precisely the person who disappears’.^25^ However, the objection that it is self‐defeating to respect autonomy by eradicating it overlooks the fact that we often find some exercises of autonomy more important than others. An example often given in this kind of context is the way Odysseus restricted his own autonomy by ordering his men to tie him to the mast of his ship. This, however, was a token of his authentic, autonomous desire not to be seduced by the song of the Sirens. Analogously, terminally ill patients may prioritise an autonomous choice regarding the manner of their death over what are, to them, less valuable choices during the final days or weeks they would otherwise live.

## 5 CONCLUSION

In this article, we have set out and critically discussed autonomy‐based arguments against the legalisation of ADR for terminally ill patients. We have shown that there are at least two variants of this type of argument. We have argued that both are problematic in light both of empirical studies and the fact that we usually believe it to be morally right to respect the wishes and decisions of dying people even if there are unbearable pain. It would be hasty to conclude that ADR ought therefore to be legalised, as there are many other arguments against ADR. Our aim has been more modest, namely, to resist a specific type of argument against the legalisation of ADR.

## CONFLICT OF INTEREST

The author declares no conflict of interest.

## Footnotes

1 See, for example, Campbell, N. (1999). A problem for the idea of voluntary euthanasia. *Journal of Medical Ethics*, *25*(3), 242–44; Hartling, O. (2021). *Euthanasia and the ethics of a doctor's decisions: An argument against assisted dying*. Bloomsbury Publishing.

2 See, for example, Hartling, O. (2021). Euthanasia and assisted dying: the illusion of autonomy—An essay by Ole Hartling. *British Medical Journal*, *374*, n2135; Campbell, J. (2005). Assisted dying and human value. *Evidence to HL Select Committee on Assisted Dying for the Terminally Ill Bill*; Heath, I. (2012). What's wrong with assisted dying. *British Medical Journal*, *344*, e3755; Sulmasy, D. P. (2017). Ethics and the psychiatric dimensions of physician‐assisted suicide. In D. A. Jones, C. Gastmans, & C. MacKellar (Eds.), *Euthanasia and assisted suicide: Lessons from Belgium* (pp. 49–64). Cambridge University Press; Velleman, J. D. (1992). Against the right to die. *Journal of Medicine and Philosophy*, *17*(6), 665–681.

3 Cohen‐Amalgor, R. (2015). First do no harm: Intentionally shortening lives of patients without their explicit request in Belgium. *Journal of Medical Ethics*, *41*(8), 625–629.

4 The description is based on Beauchamp, T. L., & Childress, J. F. (2008) *Principles of biomedical ethics*. Oxford University Press, and is very close to the wording of Petersen, T. S., & Klingenberg, R. (2022). Company‐sponsored egg freezing: An offer you can't refuse? *Bioethics*, *36*(1), 42–48.

5 Buss, S. & Westlund, A. (2018). Personal autonomy. In E. N. Zalta (Ed.), *The Stanford Encyclopedia of Philosophy*. The Metaphysics Research Lab, Stanford University.

6 Brock, D. W. (1992). Voluntary active euthanasia. *Hastings Center Report*, *22*(2), 10–22; Savulescu, J. (2015). Autonomy, interests, justice and active medical euthanasia. In M. Cholbi & J. Varelius (Eds.), *New directions in the ethics of assisted suicide and euthanasia* (pp. 41–58). Springer; Young, R. (2017). An argument in favor of the morality of voluntary medically assisted death. In M. Cholbi (Ed.), *Euthanasia and assisted suicide: Global views on choosing to end life* (pp. 167–190). Praeger.

7 It should be added, however, that one could argue that respect for autonomy should be conceived of in a broader sense according to which a patient's autonomy could still be respected even if the patient does not have the capacities to make an autonomous choice. For example, if we by mouth‐to‐mouth resuscitation save the life of a person who has had a heart failure, it seems fair to interpret such an act as one that respect the autonomy of the person. For it would respect the autonomy of the person in question, as we believe that a person who has had a heart failure would want us to bring that person back to life. However, in this article we want to focus on autonomy in a more narrow sense according to which we want to discuss whether adult terminal patients who has the capacity of autonomy and request assisted dying can be said to make autonomous choices.

8 See, for example, Young, op. cit. note 6. Countries who accept some version of assisted dying on request, do usually not accept all five conditions. For example, neither the Netherlands, Belgium or Canada accept the ‘terminal ill condition’—see for example, https://laws-lois.justice.gc.ca/eng/acts/C-46/page-33.html#h-119953. In addition, the state of California do not accept the ‘suffering condition’, see, for example, https://www.uclahealth.org/patients-families/support-information/advance-directive/introduction-california-end-life-option-act.

9 Campbell, op. cite note 1, p. 243.

10 Ibid: 242.

11 Hartling, op. cit. note 1, p. 79.

12 See, for example, van der Maas, P. J., van Delden, J. J. M., Pijnenborg, L., & Looman, C. W. N. (1991). Euthanasia and other medical decisions concerning the end of life. *Lancet*, *338*, 669–674. However, that being said, there are also studies indicating that some diseases, such as bone cancer, do inflict pain on patients that is intense and which cannot always be anticipated or removed see, for example, Krikorian, A., Limonero, J. T., & Maté, J. (2012). Suffering and distress at the end‐of‐life. *Psycho‐Oncology*, *21*(8), 799–808; Mantyh, P. W. (2014). Bone cancer pain: From mechanism to therapy. *Current Opinion in Supportive and Palliative Care*, *8*(2), 83.

13 Moen, O. M., & Sterri A. (2019). Aktiv dødshjelp: Etikk ved livets slutt (in Norwegian), Active assisted dying: Ethics at the end of life. Cappelen Damm Akademisk.

14 See, for example, Hartling op. cit. note 1.

15 Sumner, L. W. (2011). *Assisted death: A study in ethics and law*. Oxford University Press; Leigh, P. (2021). Response to euthanasia and the ethics of a doctor's decisions: An argument against assisted dying. *British Medical Journal*, *374*, n2135; Summers, D. J. (2021). Response to euthanasia and the ethics of a doctor's decisions: An argument against assisted dying. *British Medical Journal*, *374*, n2135.

16 Winzelberg, G. S., Hanson, L. C., & Tulsky, J. A. (2005). Beyond autonomy: Diversifying end‐of‐life decision‐making approaches to serve patients and families. *Journal of the American Geriatrics Society*, *53*(6), 1046–1050; Houska, A., & Loučka, M. (2019). Patients' autonomy at the end of life: A critical review. *Journal of Pain Symptom Management*, *57*(4), 835–845.

17 Heath, op. cit. note 2.

18 Hartling, op. cit. note 2, p. 1.

19 Battin, M. P., van der Heide, A., Ganzini, L., van der Wal, G., & Onwuteaka‐Philipsen, B. D. (2007). Legal physician‐assisted dying in Oregon and the Netherlands: Evidence concerning the impact on patients in “vulnerable” groups. *Journal of Medical Ethics*, *33*(10), 591–597.

20 Teisseyre, N., Mullet, E., & Sorum, P.C. (2005). Under what conditions is euthanasia acceptable to lay people and health professionals? *Social Science and Medicine*, *60*(2), 357–368.

21 Velleman, op. cit. note 2.

22 Hartling, op. cit. note, 2, p. 1.

23 Chapple, A., Ziebland, S., McPherson, A., & Herxheimer, A. (2006). What people close to death say about euthanasia and assisted suicide: A qualitative study. *Journal of Medical Ethics*, *32*(12), 706–710.

24 Hartling, op. cite. note 2, p. 1.

25 Ibid: 2.

